# Engineering Cas9: next generation of genomic editors

**DOI:** 10.1007/s00253-024-13056-y

**Published:** 2024-02-14

**Authors:** Maxim A. Kovalev, Artem I. Davletshin, Dmitry S. Karpov

**Affiliations:** 1 Engelhardt Institute of Molecular Biology, Russian Academy of Sciences, Vavilov Str., 32, 119991 Moscow, Russia; 2 Department of Biology, Lomonosov Moscow State University, 119234 Moscow, Russia; 3 Center for Precision Genome Editing and Genetic Technologies for Biomedicine, Engelhardt Institute of Molecular Biology, Russian Academy of Sciences, Vavilov Str., 32, 119991 Moscow, Russia

**Keywords:** *Streptococcus pyogenes* Cas9, Next-generation genomic editors, High-fidelity Cas9 variants, Altered PAM specificity, Cas9 immunopeptides

## Abstract

**Abstract:**

The Cas9 endonuclease of the CRISPR/Cas type IIA system from *Streptococcus pyogenes* is the heart of genome editing technology that can be used to treat human genetic and viral diseases. Despite its large size and other drawbacks, *S. pyogenes* Cas9 remains the most widely used genome editor. A vast amount of research is aimed at improving Cas9 as a promising genetic therapy. Strategies include directed evolution of the Cas9 protein, rational design, and domain swapping. The first generation of Cas9 editors comes directly from the wild-type protein. The next generation is obtained by combining mutations from the first-generation variants, adding new mutations to them, or refining mutations. This review summarizes and discusses recent advances and ways in the creation of next-generation genomic editors derived from *S. pyogenes* Cas9.

**Key points:**

• *The next-generation Cas9-based editors are more active than in the first one.*

• *PAM-relaxed variants of Cas9 are improved by increased specificity and activity.*

• *Less mutagenic and immunogenic variants of Cas9 are created.*

## Introduction

The *S. pyogenes* type II-A CRISPR/Cas9 system was the first to be harnessed for genome editing technology (Jinek et al. 2013; Cong et al. 2013; Mali et al. 2013), which is widely used in basic research and applied fields, including human gene therapy (Çerçi et al. 2023). The most commonly used CRISPR/Cas systems are type II and V of class II and are characterized by a single multidomain effector protein that has all the activities required to find the genomic target and to cleave it. *S. pyogenes* Cas9 is the most studied and widely used genomic and post-genomic editor. This is probably because Cas9 is generally more active in eukaryotic cells than other Cas effectors, both in vitro (Xin et al. 2022; Huang et al. 2023) and in vivo (Li et al. 2020). However, a number of drawbacks have hindered its use as a therapeutic genome editor, such as its large size, immunogenicity, and relatively high off-target rate compared to other Cas endonucleases. Therefore, large efforts are focused on improving the properties of *S. pyogenes* Cas9. Strategies to improve Cas9 include fusion with proteins for spatiotemporal control of Cas9 activity (Zhuo et al. 2021), fusion with proteins to add necessary functions, e.g., control of DNA repair pathways (Richardson et al. 2023), fusion with enzymes to create novel genome editors, like prime editors or base editors (Tao et al. 2023; Saber Sichani et al. 2023; Porto and Komor 2023), guide RNA engineering (Dong et al. 2022), and Cas9 engineering itself (Huang et al. 2022; Zhuo et al. 2021; Bravo et al. 2022a, b). Reviews on Cas9 engineering mainly discuss the first generation of Cas9 variants, which are derived from wild-type Cas9. First-generation Cas9 editors were created mainly to increase Cas9 fidelity. However, these variants tend to exhibit reduced on-target activity (Lee et al. 2018; Vakulskas et al. 2018; Shor et al. 2022). To address this issue, next-generation editors with not only high specificity but also increased on-target activity and other improved Cas9 characteristics are being created. This review discusses recent advances in the development of next-generation Cas9 editors in the following directions: increasing the activity of high-fidelity variants, expanding the range of targets, changing DNA repair outcome, and reducing immunogenicity.

## Structure of *S. pyogenes* Cas9 and its molecular mechanism of action

Before discussing improved Cas9 variants, it is necessary to describe the current knowledge of the structure and mechanism of action of Cas9 as an RNA-directed DNA endonuclease.

**Fig. 1:**
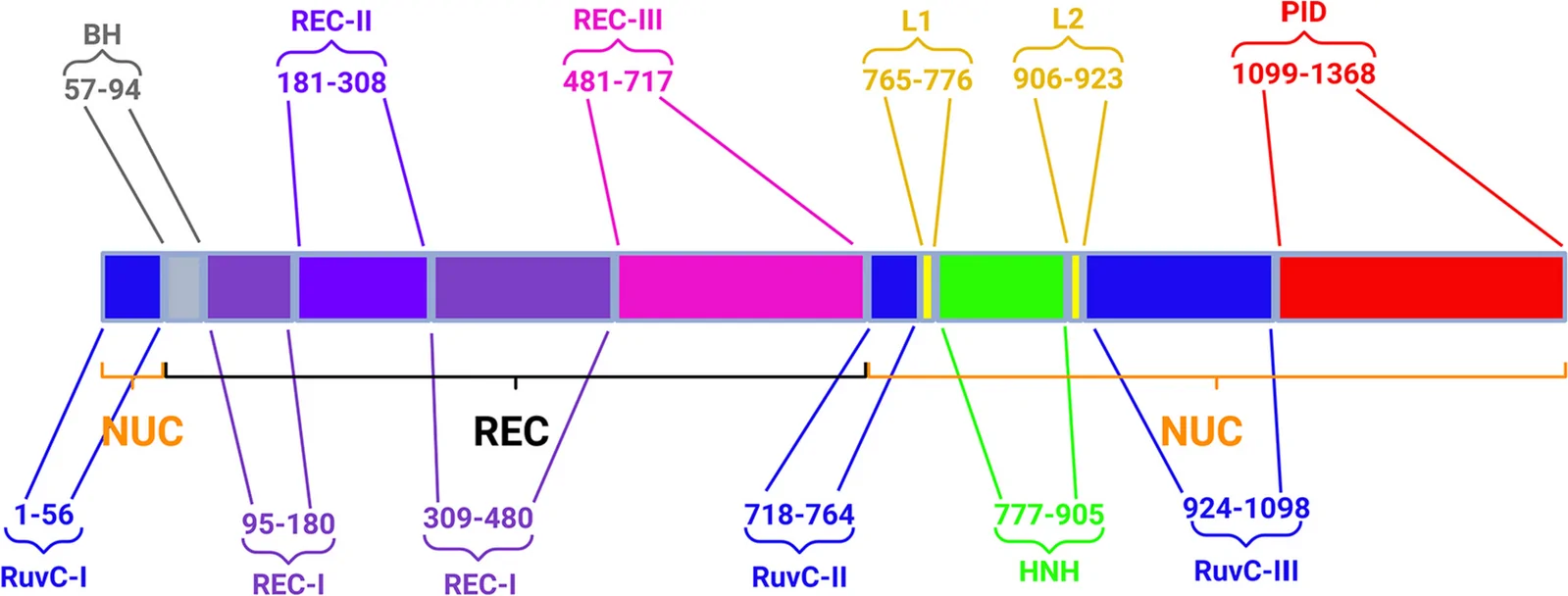
Scheme of domain positions in *S. pyogenes* Cas9. The numbers show the start and end of the domain we have accepted in this review and may vary from article to article. Abbreviations: NUC nuclease lobe, REC recognition lobe, RuvC-I, II, III nuclease domain that cleaves the non-target DNA strand, BH bridge helix, REC-I, -II, -III recognition domains, L1, L2 linkers 1 and 2, HNH nuclease domain that cleaves the target DNA strand, PID PAM-interacting domain. Created with BioRender.com

*S. pyogenes* Cas9 is a 1368 aa protein that consists of a nuclease (NUC) lobe containing two nickase domains and a recognition (REC) lobe with the ability to bind and retain a DNA-RNA duplex (Fig. 1). The most important domains of the NUC lobe are (1) RuvC, assembled from three parts of RuvC-I (1–56 aa), RuvC-II (718–764 aa), and RuvC-III (924–1098 aa), which assemble spatially and cleave the non-target DNA strand by a two-metal-dependent mechanism, (2) HNH (777–905 aa), which cleaves the target strand by a one-metal-dependent mechanism, and (3) the protospacer adjacent motif (PAM)-interacting domain (PID, 1099–1368 aa), which binds the PAM, unique to each Cas effector. The NUC lobe also includes two linkers (765–776 and 906–923 aa), which are critical for domains mobility. The REC lobe occupies positions 57–717 and consists of the REC-I (95–180 and 309–480 aa), REC-II (181–308 aa), and REC-III (481–717 aa) domains and contains the so-called bridging helix (57–94 aa), and is necessary for the formation and reorganization of the DNA-RNA complex that occurs before Cas9-dependent DNA cleavage. We emphasize that the boundaries of the corresponding domains differ slightly in different publications; therefore, we use only approximate ones here.

In the inactive apo form, Cas9 is in the open conformation and has an unstructured nucleolytic HNH domain and a protospacer interacting motif (PAM) (Jinek et al. 2014). Moreover, the active site of the HNH domain is blocked by the beta-hairpin (residues 1049–1059) of the RuvC domain (Jinek et al. 2014). The first set of major conformational changes of Cas9 occurs upon sgRNA binding that result in (1) REC-III moving in close proximity to HNH, thus displacing the inhibitory RuvC beta-hairpin and activating HNH, (2) organizing PID conformation to a state competent for PAM search, and (3) preforming the sgRNA spacer in the A-conformation, which is one of the possible modes of nucleic acid existence with 11 base pairs per turn of the right-handed helix, and such a form is thermodynamically more favorable for the formation of the sgRNA:target DNA heteroduplex, and thus it is necessary for further Cas9 action (Jiang et al. 2015). Moreover, the REC-I domain is particularly important in these motions because it contains several Glu-Glu motifs (E102–E103, E108–E109, E370–E371) that form temporary ionic bonds with positively charged residues (e.g., R69, R75, K218, K506, R635, and R1114) during these conformational changes (Liu et al. 2019). Simultaneously, the sugar-phosphate backbone of the sgRNA makes extensive contacts with the REC domains and the long arginine-rich bridging helix (residues 57–94, BH). The Cas9/sgRNA complex then searches for PAM (5′-NGG-3′) using a combination of three-dimensional and one-dimensional diffusion along the DNA (Globyte et al. 2019). R1333 and R1335 in PID are responsible for direct contacts with PAM guanines (Anders et al. 2014). However, these interactions are weak (Cofsky et al. 2022), and they are likely enhanced by contacts with the sugar-phosphate base of the sequence downstream of PAM (Zhang et al. 2019; Q. Zhang et al. 2021a, b, c), which also facilitates further steps. After binding PAM and its context, Cas9 bends and twists the target DNA with a phosphate lock loop (K1107-S1109) of PID domain and a group of four lysines (K233/K234/K253/K263), a so-called “helical core region” in the REC-II domain that binds to the sugar-phosphate backbone of the target strand (Cofsky et al. 2022). This is accompanied by a second set of lesser Cas9 conformational changes, which include relocation of the REC-II, REC-III, and HNH domains (Pacesa et al. 2022). As a result, the bases of the target strand near the PAM are flipped. If the bases of the protospacer and spacer are complementary, directional formation of the RNA:DNA hybrid, also called the R-loop, begins. R-loop formation is a rate-limiting process that is often targeted by mutations in engineered high-fidelity Cas9 variants. The ten nucleotides adjacent to the PAM (“seed region”) are critical for the formation of a stable DNA:RNA heteroduplex. Mismatches in the seed region can lead to Cas9 dissociation from the DNA fragment to probe another sequence as a target (Singh et al. 2016). The ability of Cas9 to tolerate mismatches depends largely on the free energy of formation of the RNA:DNA heteroduplex (Corsi et al. 2022). If the free energy of DNA:RNA annealing is sufficient, R-loop formation occurs in two steps (Ivanov et al. 2020). The first step is the formation of an intermediate partial R-loop, which includes mainly the seed region. The second step is the formation of the cleavage-competent open state. The success at this stage can be affected by mismatches. In general, wild-type Cas9 tolerates mismatches well outside the seed region (Zeng et al. 2018) and can even hydrolyze GC-rich targets with six mismatches although at low efficiency (Fu et al. 2013; Corsi et al. 2022). Interestingly, negative supercoiling of DNA stimulates hydrolysis of DNA targets with mismatches that are resistant to hydrolysis in the relaxed form (Ivanov et al. 2020). The observed effects can be explained by the importance for cleavage of the kinked conformation of the DNA:RNA heteroduplex characteristic of the full R-loop (Jiang et al. 2016). Thus, if the DNA:RNA heteroduplex can form a kinked conformation in the presence of mismatches, cleavage occurs. Otherwise, critical mismatches can lead to the formation of a linear DNA:RNA heteroduplex that is resistant to hydrolysis (J. P. K. Bravo et al. 2022a, b), which corresponds to the structural checkpoint of the Cas9 complex. The structural checkpoint is controlled by the REC-III domain, which senses the PAM-distal structure of the DNA:RNA hybrid (Zhu et al. 2019). If the DNA:RNA hybrid is completely annealed, the three loops in REC-III (residues 530–537, 574–588, and 686–689) become ordered and nonspecifically contact the DNA:RNA hybrid. This initiates a third large set of coordinated Cas9 rearrangements. The REC-II domain moves toward the solvent and becomes disordered. This allows the HNH domain to make an abrupt ~ 34-Å turn to take up a position against the hydrolysable phospho-diester bond of the target DNA strand. The solvent-opened loop of the RuvC domain carrying a region of positively charged residues (Lys948, Arg951, and Lys954) makes nonspecific contacts with the non-target DNA strand (Zhu et al. 2019). These concerted changes result in the positioning of the nickase domains near the PAM and the hydrolysis of the phosphodiester bonds between the third and fourth bases upstream of the PAM. RuvC cleaves the non-target DNA strand with two Mg^2+^ ions (Casalino et al. 2020), while HNH cleaves the target strand with one Mg^2+^ ion (Nierzwicki et al. 2022). Both reactions are initiated by histidines in the catalytic centers and proceed by the SN2-like mechanism. The total time required for the Cas9/sgRNA complex to hydrolyze DNA after its binding ranges from 1 to 10 min (Bisaria et al. 2017).

## Next-generation high-fidelity Cas9 variants with increased activity

**Fig. 2:**
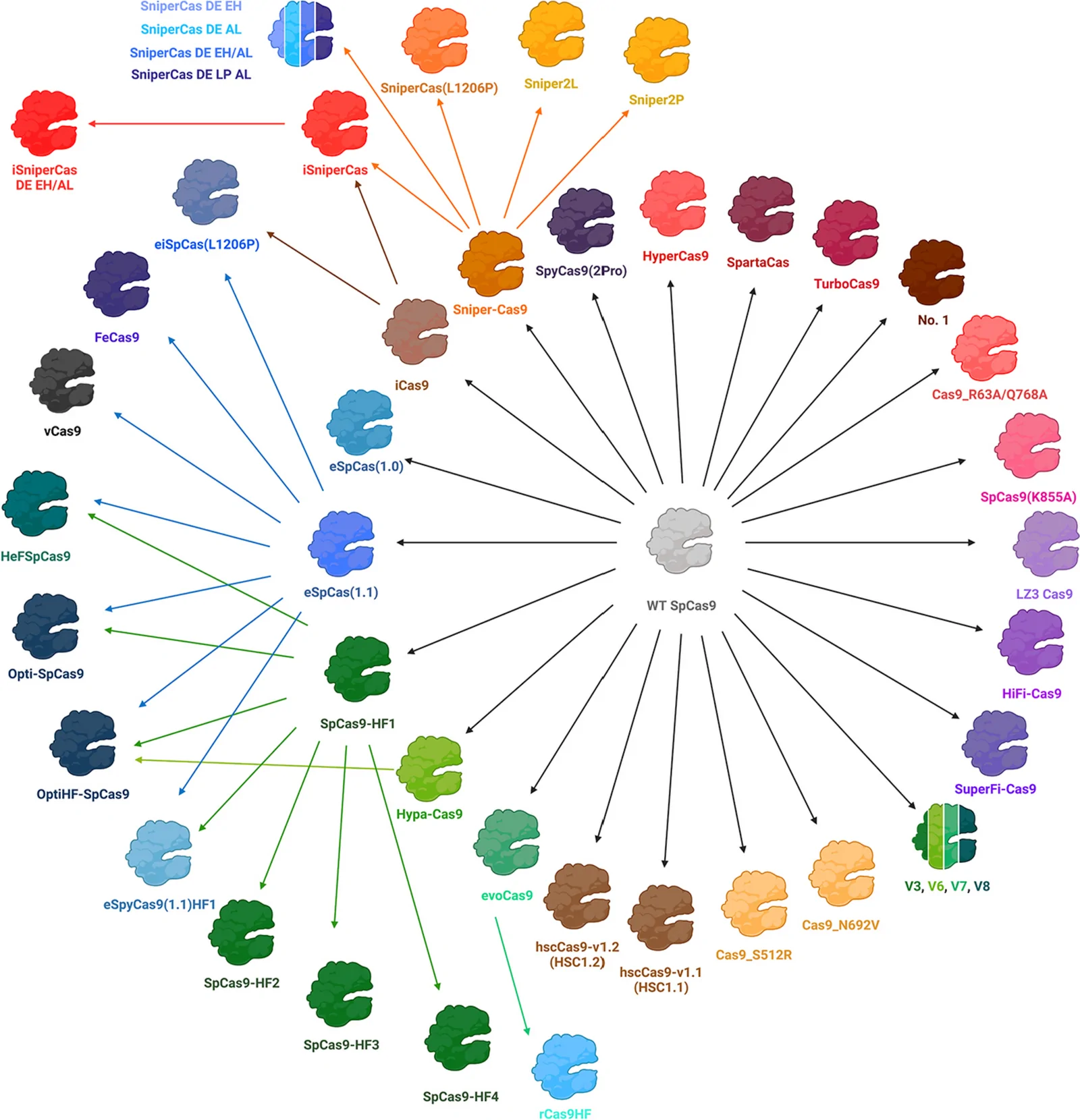
Generations of Cas9-based genome editors. First-generation editors derived directly from WT SpCas9 are shown in the inner circle; next-generation editors obtained by combining successful mutations of their precursors are shown in the outer semicircle. Created with BioRender.com

**Fig. 3:**
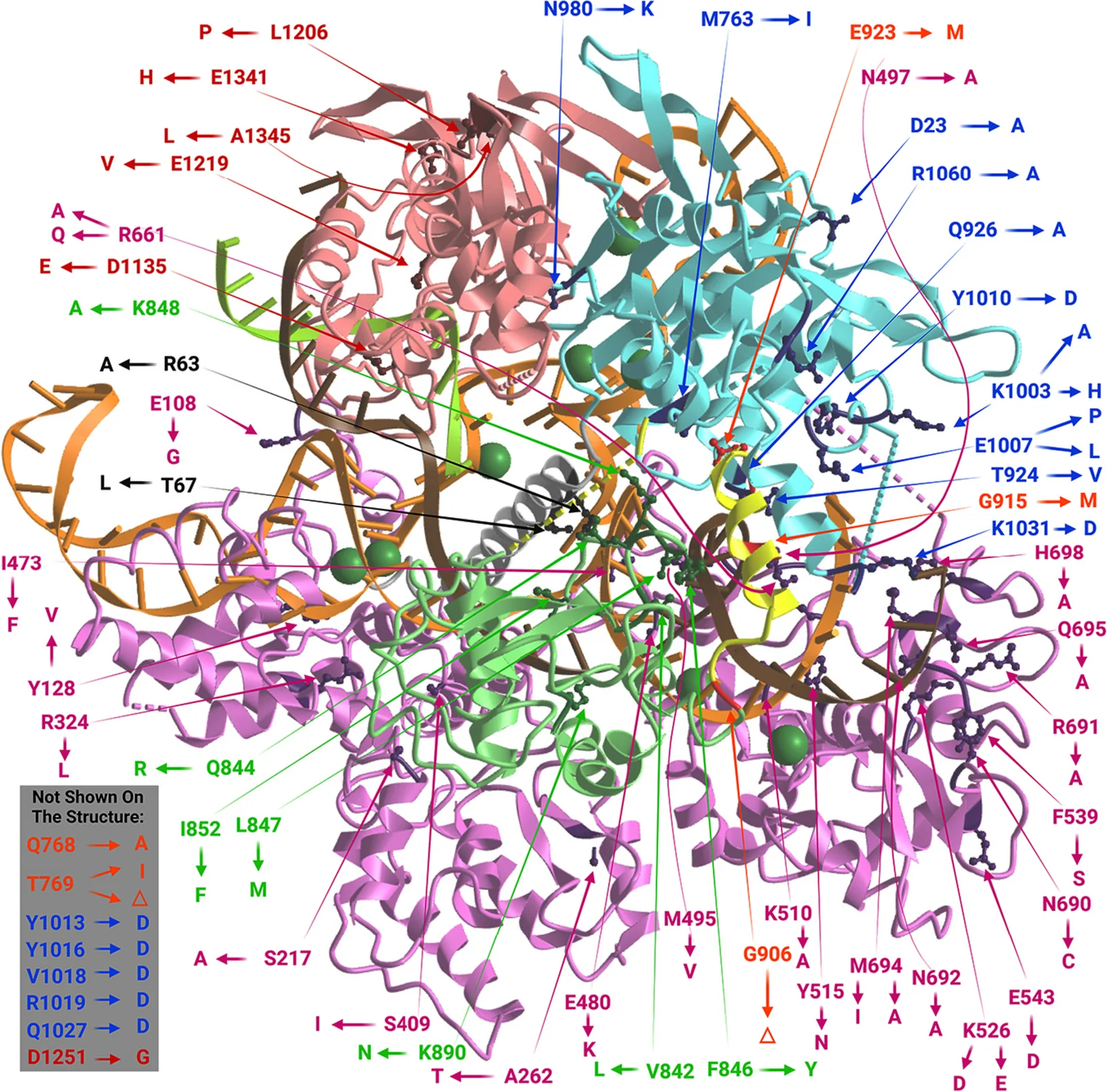
3D structure of Cas9 protein bound to nucleic acids. The structure is PDB ID 4UN3 (Anders et al. 2014). Color coding: sgRNA shown in orange, target DNA strand shown in brown, non-target DNA strand shown in light green, RuvC domain shown in light blue, BH shown in gray, REC lobe shown in lilac, linkers (L1 and L2) shown in yellow, NHN domain shown in pale green, PID shown in pink, bound magnesium ions shown in dark green. Amino acid residues the substitution of which is associated with a change in activity or specificity are shown in a brighter color and their side chains are represented as “balls and sticks”. All of them (including those missing on the 4UN3 structure) are signed indicating which amino acids they were replaced with to create improved SpCas9-based editors, with the colors of the signatures corresponding to the domains to which these residues belong. The initial picture was created using icn3d (Wang et al. 2022). The final version was created with BioRender.com

The evolutionary advantage of Cas9’s ability to fight bacteriophages, despite the presence of single mutations in the target sequences, becomes a disadvantage for genome editing technology. The main feature of the first-generation of genomic editors derived from wild-type Cas9 (Figs.2 and 3, Table 1) is increased specificity. Specificity can be increased in several ways. For example, the first highly specific variant of eSpCas9(1.1) was engineered by reducing the nonspecific binding of the HNH, RuvC-III, and PID domains to the non-target DNA strand to facilitate reverse DNA strand annealing in case of mismatches between the spacer and the target strand (Slaymaker et al. 2016). The next variant of Cas9-HF1 was obtained by disrupting nonspecific contacts with the target DNA strand by mutating residues in the REC-III and RuvC-III domains (Kleinstiver et al. 2016). However, one of the costs of increased specificity is reduced on-target activity. It turned out that K848 is a key residue for sumoylation of Cas9, which protects the protein from polyubiquitination and subsequent proteasome-dependent degradation (Ergünay et al. 2022). Thus, the K848 mutation in eSpCas9 (1.1) negatively affects Cas9 activity through enhanced Cas9 degradation. So, decreased Cas9 levels may be one of the reasons for decreased off-target as well as on-target activity. It is also likely that combinations of mutations in first-generation genomic editors can disrupt the structure of Cas9 domains and thereby make Cas9 mutants susceptible to proteasome-dependent degradation.

**Table 1:** Cas9-derived genomic editors with improved specificity and activity

Cas9 variant	Mutations	Reference
First generation of Cas9 editors		
eSpCas (1.0)	K810A, K1003A, R1060A	(Slaymaker et al. 2016)
eSpCas (1.1)	K848A, K1003A, R1060A	
SpCas9 (K855A)	K855A	
Cas-HF1	N497A, R661A, Q695A, Q926A	(Kleinstiver et al. 2016)
Hypa-Cas9	N692A, M694A, Q695A, H698A	(J. S. Chen et al. 2017a, b)
evoCas9	M495V, Y515N, K526E, R661Q	(Casini et al. 2018)
iCas9	D147Y, P411T	(Bao et al. 2015)
xCas9 (3.6)	E108G, S217A, S409I, E480K, E543D, M694I, E1219V	(Hu et al. 2018)
xCas9 (3.7)	A262T, R324L, S409I, E480K, E543D, M694I, E1219V	
Sniper-Cas9	F539S, M763I, K890N	(Lee et al. 2018)
HyperCas9	I473F	(Heler et al. 2017)
SpyCas9 (2Pro)	L64P, K65P	(Babu et al. 2019)
Cas9_R63A/Q768A	R63A, Q768A	(Bratovič et al. 2020)
SuperFi-Cas9	Y1010D, Y1013D, Y1016D, V1018D, R1019D, Q1027D, K1031D	(J. P. K. Bravo et al. 2022a, b)
Turbo Cas9	V842L, Q844R, F846Y, L847M, I852F	(Vos et al. 2022)
HiFi-Cas9	R691A	(Vakulskas et al. 2018)
SpartaCas	D23A, T67L, Y128V, D1251G	(Cerchione et al. 2020)
LZ3 Cas9	N690C, T769I, G915M, N980K	(Schmid-Burgk et al. 2020)
hscCas9-v1.1 (HSC1.1)	N588A, R765A, D835A, K1246A	(Zuo et al. 2022)
hscCas9-v1.2 (HSC1.2)	N14A, R447A, R765A, S845D	
Cas9_S512R	S512R	(Rabinowitz et al. 2023)
Cas9_N692V	N692V	
V3	N497A, K510A, R661A	(Wang et al. 2024)
V6	K510A	(Wang et al. 2024)
V7	N497A, K510A	(Wang et al. 2024)
V8	K510A, R661A	(Wang et al. 2024)
“No. 1”	△T769, △G906	(Wang et al. 2024)
Next-generation Cas9 editors		
Cas-HF2	N497A, R661A, Q695A, Q926A, D1135E	(Kleinstiver et al. 2016)
Cas-HF3	L169A, N497A, R661A, Q695A, Q926A,	
Cas-HF4	Y450A, N497A, R661A, Q695A, Q926A,	
HeFSpCas9	N497A, R661A, Q695A, K848A, Q926A, K1003A, R1060A	(Kulcsár et al. 2017)
FeCas9	K848A, K1003A, R1060A, D1135E	(Yin et al. 2019)
Opti-SpCas9	R661A, K1003H	(Choi et al. 2019)
OptiHF-SpCas9	Q695A, K848A, E923M, T924V, Q926A	
rCas9HF	K526D	(Pedrazzoli et al. 2023)
eiSpCas (L1206P)	D147Y, P411T, K848A, K1003A, R1060A, L1206P	(Spasskaya et al. 2023)
SniperCas (L1206P)	F539S, M763I, K890N, L1206P	
iSniperCas	D147Y, P411T, F539S, M763I, K890N	(Spasskaya et al. 2023)
SniperCas DE EH	F539S, M763I, K890N, D1135E, E1341H	(Davletshin et al. 2024)
SniperCas DE AL	F539S, M763I, K890N, D1135E, A1345L	(Davletshin et al. 2024)
SniperCas DE LP AL	F539S, M763I, K890N, D1135E, L1206P, A1345L	(Davletshin et al. 2024)
iSniperCas DE EH/AL	D147Y, P411T, F539S, M763I, K890N, D1135E, E1341H, A1345L	(Davletshin et al. 2024)
Sniper2L	F539S, M763I, K890N, E1007L	(Kim et al. 2023)
Sniper2P	F539S, M763I, K890N, E1007P	
vCas9	S55R, R976A, K1003A, T1314R	(Chauhan et al. 2023)

Another big price for increased specificity is the slowed kinetics of DNA hydrolysis (M. S. Liu et al. 2020a, b; Jones et al. 2021). Unexpectedly, despite the decrease in nonspecific DNA contacts, the high-fidelity variants showed DNA binding affinity at the level of wild-type Cas9 (Jones et al. 2021). Thus, the decrease in the rate of DNA cleavage is probably due to a change in post-binding events. Conformational dynamics studies suggest a strengthening of the structural checkpoint that significantly slows the transition to the catalytically active state in high-fidelity Cas9 variants (Yang et al. 2018).

We next discuss examples of two papers in which hypothesis-driven first-generation genomic editors with increased specificity while retaining targeting activity were developed. In the first paper, the authors formulated the “HH theory”, according to which the sgRNA:DNA hybrid is extruded, which leads to enhanced hydrophobic interactions between the hybrid and REC-III/HNH, ultimately triggering cleavage initiation. Thus, many mutations in known high-fidelity editors (e.g., SpCas9-HF1, eSpCas9 (1.1), HypaCas9) lead to a reduction in nonspecific contacts between the hybrid and the protein, allowing only perfect interactions to trigger cleavage (G. Wang et al. 2021). Using HH theory as a theoretical framework, they selected several amino acid residues that form strong hydrophobic interactions with the duplex. Replacement of Lys510, Asn497, and Arg661 with alanine in various combinations yielded novel V3, V6, V7, and V8 editors with increased specificity and virtually no loss of activity compared to WT (Wang et al. 2024).

In the second paper, a “No. 1” variant carrying deletions in the L1 and L2 linkers (△Thr769 and △Gly906, respectively) was obtained. The rationale for this was the “energy-distance hypothesis”, according to which the energy of sgRNA:DNA hybrid formation is spent on moving the HNH domain, and the more critical the mismatches are, the smaller this energy is. Therefore, lengthening the distance from the HNH to the hybrid by shortening the linkers should have allowed only the perfectly matched target to be cut. Indeed, the resulting form had fidelity comparable to HypaCas9 and eSpCas9 (1.1) and was superior to HypaCas9 in terms of on-target activity, while many other forms with linker deletions near the NHN (L1 and L2) and REC-III domains were partially or completely inactivated, suggesting the need for caution when constructing genomic editors using this approach (Wang et al. 2023).

Next-generation Cas9 editors with increased on-target activity have been rationally constructed by re-examining the original mutations based on novel Cas9:sgRNA:DNA structures and results from molecular modeling experiments or using protein evolution with particular attention to activity. For example, Sniper2L/2P variants were derived from Sniper-Cas9 (Sniper1) using protein evolution with saturation mutagenesis of identified new sites (Kim et al. 2023). Another example is rCas9HF derived from evoCas9 (Pedrazzoli et al. 2023). evoCas9 is the most specific yet weakest variant of Cas9 (Schmid-Burgk et al. 2020), carrying four mutations in the REC-III domain (Table 1). The authors found that combinations of triple or double mutations also reduced activity. Perhaps the combination of several mutations in the same domain negatively affects its structure, increasing proteasome-dependent degradation. Thus, the authors characterized single mutations and found that K526E increases specificity and has no significant effect on activity. A further round of mutagenesis focusing on K526 led to the identification of K526D, which increases specificity while maintaining activity at the level of wild-type Cas9 (Pedrazzoli et al. 2023). At present, the mechanism is not fully understood, but this variant can be considered a good platform for further Cas9 engineering.

Another way for improving first-generation Cas9 editors was implemented in our recent work by adding a novel L1206P mutation in PID (Spasskaya et al. 2023). The mutation position has not been previously identified in any work, including the comprehensive Cas9 mutagenesis screening (Spencer and Zhang 2017). Depending on the Cas9 variant, this mutation can increase activity while maintaining high specificity. At first glance, it is not clear how mutations in PID can affect Cas9 activity. However, target recognition is initiated by PAM binding, and therefore mutations affecting this key step can change Cas9 activity in general. Moreover, our molecular modeling studies have also indicated that there may be long-range intramolecular interactions, and L1206P also affects the structure of the RuvC active center. This interaction is possible through the RuvC interface contacting PID. Thus, the L1206P mutation may have multiple effects on Cas9 activity. Further studies led to the discovery of two more amino acid residues, E1341 and A1345, spatially close to L1206, whose mutations, E1341H and A1345L, can restore the activity of highly specific forms of SpCas9, such as SniperCas DE and iSniperCas DE. Thus, a cluster of amino acid residues was found in PID, mutations in which are able to increase the activity of highly specific Cas9 variants (Davletshin et al. 2024).

A growing body of evidence suggests that the nucleosome presents a barrier to genome editing (Verkuijl and Rots 2019; Dubois 2022). This barrier appears to be higher for high-fidelity Cas9 variants (X. Chen et al. 2017a, b; Spasskaya et al. 2023). Therefore, several ways to overcome this barrier are being explored, including recruiting a transcriptional machinery (Liu et al. 2019; Daer et al. 2020), adding DNA- or histone-binding proteins and peptides (Ding et al. 2019), or using inhibitors of chromatin-modifying complexes (B. Liu et al. 2020a, b; J. P. Zhang et al. 2021a, b, c). Cas9 engineering may represent a compact and promising strategy. The first variant to show increased activity in the context of yeast chromatin was iCas9 carrying D147Y and P411T mutations in the REC-I domain (Bao et al. 2015). Our work shows that the addition of iCas9 mutations generally increases the activity of high-fidelity variants (Spasskaya et al. 2023). However, off-target activity is also increased. Another pair of R221K and N394K mutations in the REC-II and REC-I domains, respectively, increased the activity of the chimeric iSpyMAC (a Cas9-based editor for the AA dinucleotide PAM) (Chatterjee et al. 2020a, b). The L1206P or A1345L mutations also enhance the activity of wild-type Cas9 and its high-fidelity derivatives on nucleosomes (Spasskaya et al. 2023; Davletshin et al. 2024). Clearly, Cas9 mutations in different domains suggest that the nucleosome barrier can be overcome by different mechanisms. Our current knowledge suggests that the L1206P mutation may indirectly increase PAM binding in the chromatin context, an important step to win competition with histones for target recognition (Hinz et al. 2015; Yarrington et al. 2018). How mutations in the REC domains contributed to increased Cas9 activity in the chromatin context is currently unclear.

## Expanding the range of targets: overcoming PAM limitations

Despite the short PAM (actually a GG dinucleotide), the range of possible genomic targets of Cas9 is considerably limited. Based on the occurrence of the GG dinucleotide, wild-type Cas9 is able to bind approximately 1/16 of all possible genomic targets. Cas9 is also capable of recognizing PAM NAG, NGA (Jiang et al. 2013; Zhang et al. 2014) and NHGG (Collias et al. 2020) with less efficiency. And yet, a large number of targets, for example, in AT-rich regions of regulatory and protein-coding regions of genes remain inaccessible. Therefore, a direction in Cas9 engineering related to the relaxation of PAM-dependence is developing (Collias and Beisel 2021). This direction can be divided into two parts: (1) reducing PAM requirements toward making PAM-free enzymes and (2) creating Cas9 variants that recognize novel PAM sequences.

The first attempt to relax PAM recognition was the creation of several variants named after the amino acid substitutions they carry: SpCas9-VQR and SpCas9-VRQR both recognize NGA PAM, SpCas9-EQR binds NGAG, and SpCas9-VRER prefers NGCG sequence (Kleinstiver et al. 2015, 2016). Subsequently, a series of xCas9 variants, including xCas9(3.6) and xCas9(3.7) with recognition of NG and some other PAMs and increased specificity but decreased activity, emerged from a protein evolution experiment (Hu et al. 2018). Later, a more active variant of SpCas9-NG was obtained (Nishimasu et al. 2018), which, however, was outperformed by a form of SpG with the same specificity to NGN PAM (Walton et al. 2020). This served as the basis for the creation of the SpRY mutant, which used to be the closest to the PAM-free nuclease concept. It has a weak preference for NRN PAMs, interacting more readily with them than with NYN ones (Walton et al. 2020). The SpdCas9NG-QT and SpdCas9NG-LWQT variants show a PAM recognition profile similar to that of SpRY (J. Wang et al. 2021).

The most “PAM-free” Cas9 variant to date is SpRYc. The premise for its creation was the discovery of an ortholog of SpCas9 from *S. canis* (ScCas9) that recognizes NNG PAM, and its ignoring of the base at the second position is due to an important structural feature, a positively charged loop (IKHRKRKRTTKL) at positions 367–376, which is absent in other Cas9 orthologs and presumably originated by insertion (Chatterjee et al. 2018). Its further refinement, including increasing the positive charge on this loop, resulted in the Sc ++ variant (Chatterjee et al. 2020a, b). Finally, grafting the PID (residues 1111–1368) of SpRY to the N-terminus (residues 1–1119) of Sc ++ resulted in the SpRYc protein with a total length of 1377. Like its predecessors, SpRYc is not entirely PAM-free, but against those PAMs on which SpRY exhibits almost zero activity (e.g., NTA and NTT), SpRYc performs ~ 10–400 times better, allowing it to be used to edit previously inaccessible regions of the genome, and it is the positively charged loop from Sc ++ that reduces the requirement of this editor for the second position of PAMs. Interestingly, because Sc ++ is an incredibly precise enzyme, SpRYc has less pronounced off-target activity than SpRY, although it is outperformed by WT SpCas9 (Zhao et al. 2023).

The story of Cas9 nucleases with altered PAM specificity includes multiple SpCas9-NRRH, SpCas9-NRTH, and SpCas9-NRCH mutants that recognize NRRH, NRTH, and NRCH PAM, respectively (Miller et al. 2020), QQR1 with specificity to NAAG (Anders et al. 2016), KG and VRKG with increased NAG and decreased NGG affinity (Goldberg et al. 2021). On the other hand, the D1135E mutation reduces activity on NAG and NGA PAMs while retaining it on NGG and NHGG PAMs (Collias et al. 2020). A hybrid approach was applied once again by Chatterjee et al.: the authors replaced the *S. pyogenes* Cas9 PID with a PID from *Streptococcus macacae* Cas9, which has a natural affinity to NAAN PAMs (Chatterjee et al. 2020a, b).

The effect of some mutations on PAM specificity is not always understood. We will briefly discuss mutations whose mechanism of action is clear. So, the mechanism of direct action of substitutions R1333 or R1335, which are necessary for G2 or G3 recognition in NGG PAM, respectively, is understood. Usually, such mutants lose G specificity at the corresponding position: R1333G (as in KG and VRK) and R1335P (as in SpRY) eliminate all specific interactions, and R1333Q or R1335Q favors A over G, as in QQR1 (Goldberg et al. 2021; Walton et al. 2020; Anders et al. 2016). On the other hand, the T1337R substitution results in a slight preference for 4G because R1337 can interact with DNA similarly to R1333 and R1335 (Anders et al. 2016). Other mutations alter specificity indirectly. For example, E1219V is the only xCas9(3.7) mutation that alters PAM because E1219 stabilizes R1335 binding to G3 by forming a salt bridge (Guo et al. 2019). Some mutations are necessary to compensate for the loss of the specific interaction. For example, D1332K in KG, VRKG, and probably QQR1 facilitates nonspecific interaction with the sugar-phosphate backbone of the target DNA chain (Goldberg et al. 2021). The D1135V mutation in the VQR, VRQR, and VRER variants results in the loss of the local negative charge and thereby increases affinity for the non-target DNA strand (Anders et al. 2016). Not all mutations affecting PAM specificity are localized in PID: for example, in recent work, an additional P411T substitution was introduced into xCas9 (3.7), which resulted in a change in the dynamic properties of the REC-I domain and the protein became more active in recognizing AGT, ACG, GAC, and NAC PAMs (Liu et al. 2022). Therefore, even changing distant positions in the Cas9 protein with other targets can change its PAM specificity, which, of course, should be taken into account when designing improved genomic editors. In conclusion, we emphasize that understanding the role of individual mutations and their interactions in the case of PAM recognition is absolutely essential for the rational design of novel PAM-relaxed or PAM-modified Cas9 variants.

**Fig. 4:**
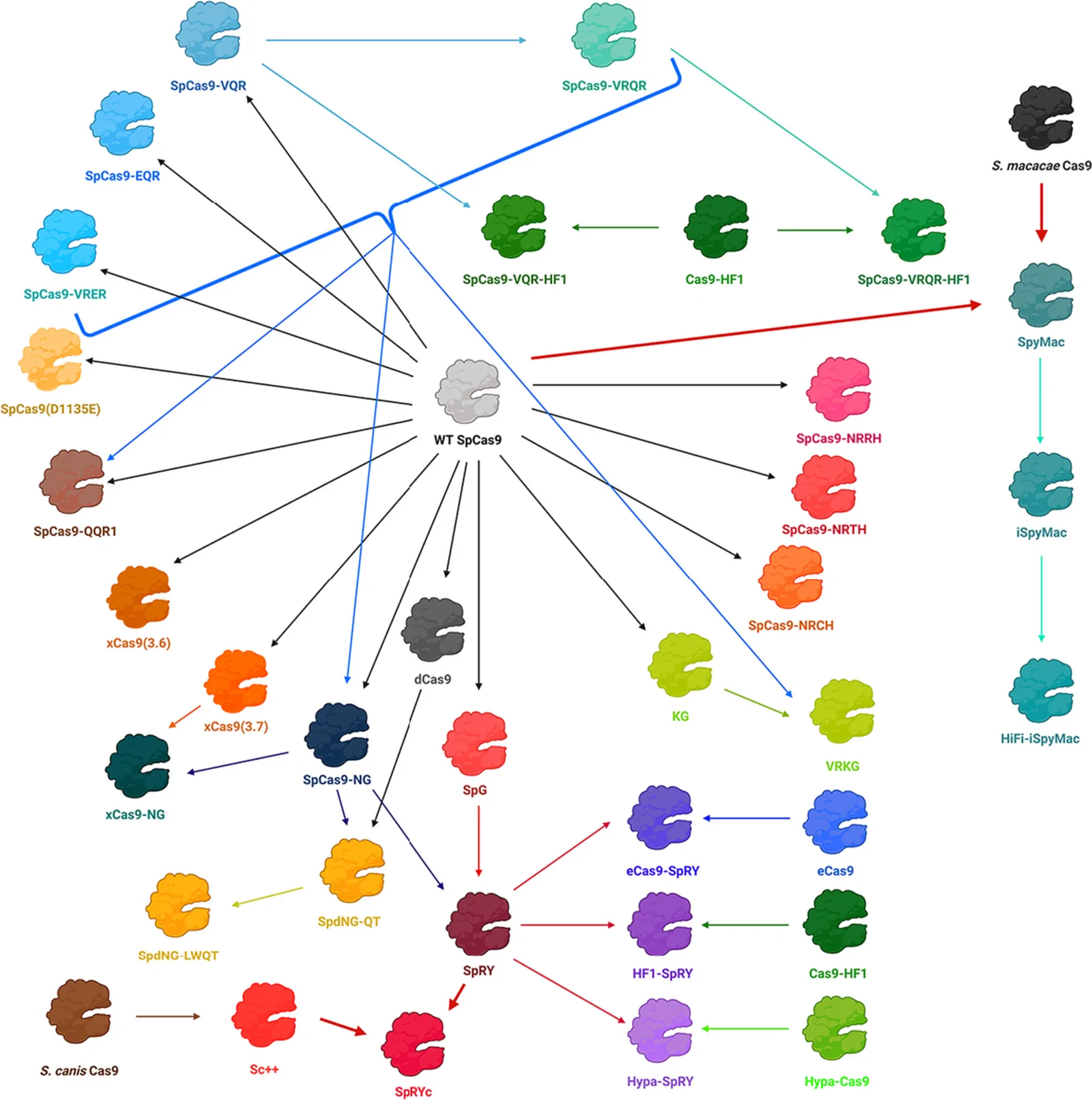
Development of Cas9 variants with relaxed or altered PAM recognition. Mutational relationships are marked with arrows. *S. macacae* Cas9 and Sc ++ became domain donors for creation of chimeric proteins SpyMac and SpRYc, respectively, as indicated by bold red arrows. The colored thin arrows show the transformations of already modified SpCas9 variants. Created with BioRender.com

**Fig. 5:**
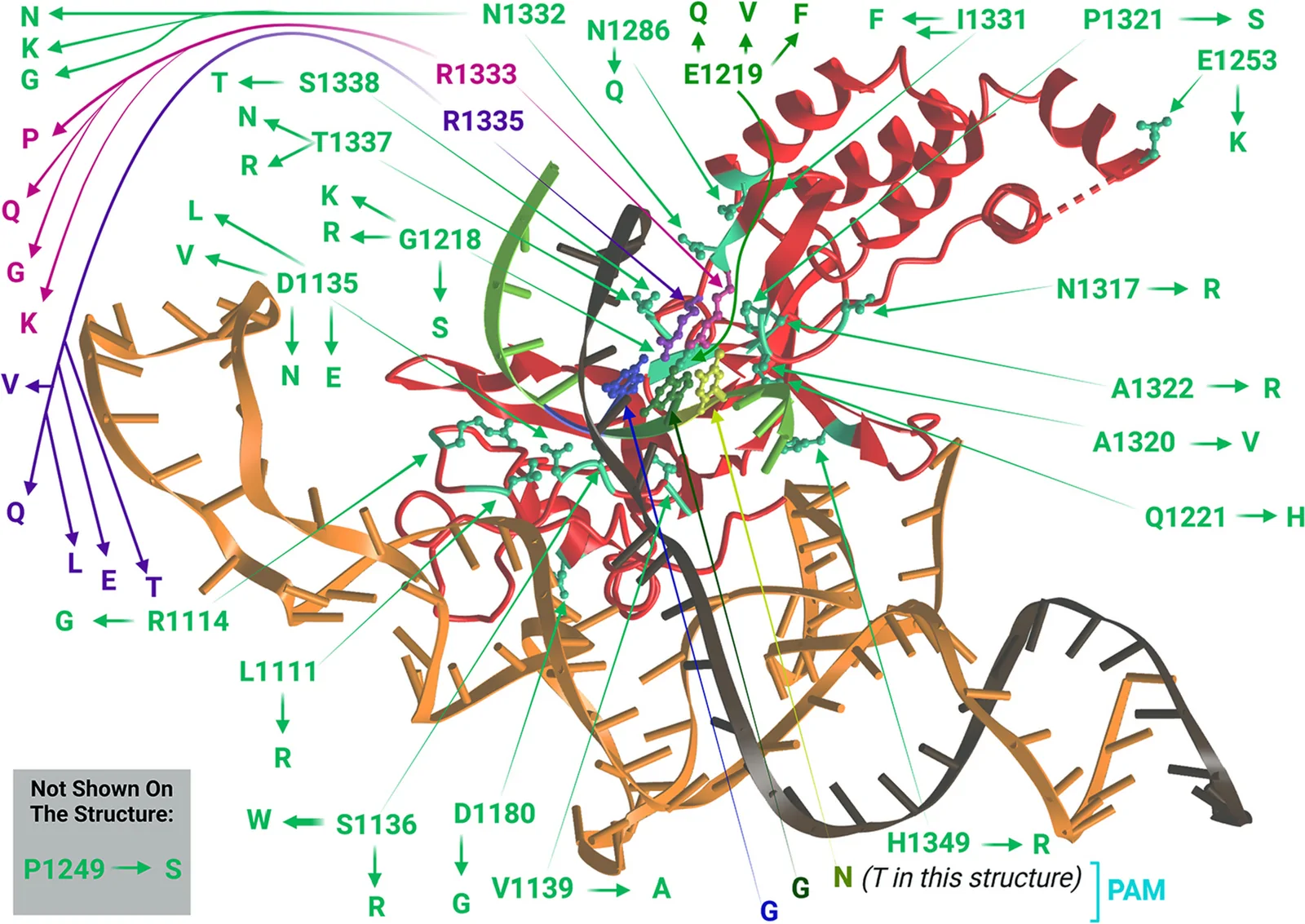
3D structure of Cas9 protein PAM-interacting domain bound to nucleic acids. The structure is PDB ID 4UN3 (Anders et al. 2014). Color coding: sgRNA shown in orange, target DNA strand shown in brown, non-target DNA strand shown in light green, PID domain shown in red, other domains and bound magnesium ions absent. Amino acid residues the substitution of which is associated with a change in PAM specificity are shown in mint, R1333 is shown in magenta, R1335 is shown in purple, their side chains are represented as “balls and sticks”. Similarly, the constituent nucleotides of PAM are differentially colored: N (~ T) is shown in yellow; G is shown in green; next G is shown in blue; their bases are represented as “balls and sticks”. All of them (including one missing on the 4UN3 structure) are signed indicating which amino acids they were replaced with to create SpCas9-based editors with altered PAM specificity. The initial picture was created using icn3d (Wang et al. 2022). The final version was created with BioRender.com

To summarize, the currently known Cas9 variants are presented in Table 2, their relatedness is shown in Fig. 4, and the 3D structure of PID with bound nucleic acids with marked amino acid residues the substitution of which leads to a change in PAM specificity is shown in Fig. 5. Taken together, the currently known Cas9 variants potentially cover significantly more than the 56% of all genomic targets previously evaluated (Collias & Beisel 2021). Cas9 modifications affect mainly PAM-recognizing arginines (R1333 and R1335), residue E1219, which mutation provides increased conformational mobility for R1335 (Chen et al. 2019; Guo et al. 2019), and residues located in close spatial proximity to and affecting PAM-recognizing arginines and that can also form contacts with other nucleotides.

**Table 2:** Cas9 variants with relaxed or altered PAM recognition

Cas9 variant	Mutations	Recognized PAM(s)	Reference
WT SpCas9	-	NGG > NGA, NAG, N(A/C/T)GG	(Jiang et al. 2013; Zhang et al. 2014; Collias et al. 2020)
SpCas9-VQR	D1135V, R1335Q, T1337R	NGA	(Kleinstiver et al. 2015)
SpCas9-EQR	D1135E, R1335Q, T1337R	NGAG	
SpCas9-VRER	D1135V, G1218R, R1335E, T1337R	NGCG	
SpCas9-VRQR	D1135V, G1218R, R1335Q, T1337R	NGA	(Kleinstiver et al. 2016)
SpCas9-VQR-HF1	N497A, R661A, Q695A, Q926A, D1135V, R1335Q, T1337R	NGA	
SpCas9-VRQR-HF1	N497A, R661A, Q695A, Q926A, D1135V, G1218R, R1335Q, T1337R	NGA	
SpCas9(D1135E)	D1135E	NGG and N(C/T/A)GG, reduced recognition of NGA and NAG	(Collias et al. 2020)
QQR1	G1218R, N1286Q, I1331F, D1332K, R1333Q, R1335Q, T1337R	NAAG	(Anders et al. 2016)
xCas9(3.6)	E108G, S217A, S409I, E480K, E543D, M694I, E1219V	NG, NNG, GAA, GAT, CAA	(Hu et al. 2018)
xCas9(3.7) ~ xCas9	A262T, R324L, S409I, E480K, E543D, M694I, E1219V	NG, NNG, GAA, GAT, CAA	(Hu et al. 2018; Guo et al. 2019)
SpCas9-NG (VRVRFRR)	R1335V, L1111R, D1135V, G1218R, E1219F, A1322R, T1337R	NG	(Nishimasu et al. 2018)
xCas9-NG	A262T, R324L, S409I, E480K, E543D, M694I, L1111R, D1135V, G1218R, E1219F, A1322R, R1335V, T1337R	NG	(Legut et al. 2020)
SpG	D1135L, S1136W, G1218K, E1219Q, R1335Q, T1337R	NGN	(Walton et al. 2020)
SpRY	A61R, L1111R, D1135L, S1136W, G1218K, E1219Q, N1317R, A1322R, R1333P, R1335Q, T1337R	NRN	
SpRYc	N-terminus (1–1119) from Sc ++ and PID (1111–1368) from SpRY	NNN	(Zhao et al. 2023)
SpCas9-NRRH	D10T, I322V, S409I, E427G, R654L, R753G, R1114G, D1135N, V1139A, D1180G, E1219V, Q1221H, A1320V, R1333K	NRRH	(Miller et al. 2020)
SpCas9-NRTH	D10T, I322V, S409I, E427G, R654L, R753G, R1114G, D1135N, D1180G, G1218S, E1219V, Q1221H, P1249S, E1253K, P1321S, D1332G, R1335L	NRTH	
SpCas9-NRCH	D10T, I322V, S409I, E427G, R654L, R753G, R1114G, D1135N, E1219V, D1332N, R1335Q, T1337N, S1338T, H1349R	NRCH	
SpdNG-QT	D10A, H840A, R1335V, L1111R, G1218R, E1219F, A1322R, R1333Q, V1335T, T1337R	NRN and some NYN. PAM profile is similar to SpRY, with a stronger affinity for NRT (including CAT) and NGG PAMs	(J. Wang et al. 2021)
SpdNG-LWQT	D10A, H840A, R1335V, L1111R, D1135L, S1136W, G1218R, E1219F, A1322R, R1333Q, V1335T, T1337R	NRN and some NYN. PAM profile is similar to SpRY, with a stronger affinity for NRT (including CAT) and NGG PAMs	
KG	D1332K, R1333G	NAG	(Goldberg et al. 2021)
VRKG	D1135V, S1136R, D1332K, R1333G	NAG	
eCas9-SpRY	A61R, K848A, K1003A, R1060A, L1111R, D1135L, S1136W, G1218K, E1219Q, N1317R, A1322R, R1333P, R1335Q, T1337R	NRN	(W. Zhang et al. 2021a, b, c)
HF1-SpRY	A61R, N497A, R661A, Q695A, Q926A, L1111R, D1135L, S1136W, G1218K, E1219Q, N1317R, A1322R, R1333P, R1335Q, T1337R	NRN	
Hypa-SpRY	A61R, N692A, M694A, Q695A, H698A, L1111R, D1135L, S1136W, G1218K, E1219Q, N1317R, A1322R, R1333P, R1335Q, T1337R	NRN	
SpyMac	PID from *S. macacae* Cas9	NAAN	(Chatterjee et al. 2020a, b)
iSpyMac	PID from *S. macacae* Cas9, R221K, N394K	NAAN	
HiFi-iSpyMac	PID from *S. macacae* Cas9, R221K, N394K, R691A	NAAN

The relaxation of PAM recognition gives rise to a number of drawbacks, overcoming which leads to the creation of a new generation of editors. The first disadvantage is increased off-target activity (W. Zhang et al. 2021a, b, c). Moreover, in the absence of PAM, the CRISPR/Cas9 system would not be able to distinguish between alleles of genes with nearly identical sequences (Rabinowitz and Offen 2021). Known solutions include adding new mutations to the REC domains to increase the fidelity of PAM-relaxed Cas9 variants (Chen et al. 2019) or mutations from known SpCas9 variants with high fidelity to produce chimeras such as eCas9-SpRY, HF1-SpRY, and Hypa-SpRY (W. Zhang et al. 2021a, b, c) or SpCas9-VQR-HF1 and SpCas9-VRQR-HF1 (Kleinstiver et al. 2016). A second disadvantage is the increasing risk of autotargeting of the vector expressing sgRNA, which reduces the activity of the system. Interestingly, this issue was not observed in the case of SpRYc, the most “PAM-free” SpCas9 derivative (Zhao et al. 2023). This can be overcome by using a modified sgRNA base structure. For example, in Qin et al. (2020), the authors suggested using the sgRNA scaffold starting with 5′-GCCCC-3′. Another solution is to use PAM-relaxed variants, such as SpdNG-LWQT with dCas9 devoid of nuclease activity, for epigenetic applications (J. Wang et al. 2021). The third drawback is a decrease in on-target activity (W. Zhang et al. 2021a, b, c; Legut et al. 2020), which could also be the result of more than just auto-targeting. Although Cas9 weakly contacts PAM (Cofsky et al. 2022), it is possible that conformational changes in PID during PAM binding may induce nonspecific post-PAM interactions that enhance Cas9 binding to a potential target. Reducing or abolishing PAM interactions may reduce the strength of post-PAM interactions and thereby reduce Cas9’s ability to unwind DNA. Another data set suggests that interactions with PAM (and possibly its context) are critical for Cas9 to win competition with histones for protospacer interactions (Hinz et al. 2015; Handelmann et al. 2023). Therefore, weakening the interaction of Cas9 with PAM and its context would significantly reduce its ability to edit DNA in the chromatin context. Moreover, it is conceivable that decreased specificity may also contribute to decreased Cas9 activity by enhancing Cas9 delocalization to extra-genomic targets. Known solutions for enhancing the activity of PAM-engineered Cas9 variants include: (1) a combination of known mutations, as in xCas9-NG, which combines mutations from xCas9 (3.7) and Cas9-NG variants (Legut et al. 2020), (2) structure-directed design of mutations in additional residues that have no direct contacts with PAM but are nevertheless important for PAM recognition or non-specific contacts with DNA (Walton et al. 2020), (3) introduction of Cas9-activating mutations R221K and N394K in the REC-I domain (Chatterjee et al. 2020a, b). Thus, the creation of a PAM-independent Cas9 editor requires the introduction of a combination of additional mutations that restore its specificity and activity.

## Changing the DNA repair outcome: formation sticky ends instead of blunt ends

An emerging direction of Cas9 engineering is to reduce its mutagenicity by controlling the structure of double-strand breaks (DSBs). The structure of DSBs is one of the factors determining the choice of the operating DNA repair pathway. Blunt DNA ends, that is, ends of a DNA molecule that lack any overhanging sequences, are the predominant substrates for the non-homologous end joining (NHEJ) pathway, which is a DNA repair mechanism that joins two broken, usually closely spaced, ends of DNA together, without using a homologous template for this process. At the same time, DNA ends with short overhangs of ssDNA are substrates for microhomology-mediated end joining (MMEJ) pathway, which involves the annealing of short (5–25 base pairs) homologous sequences, or microhomologies, between the single-stranded overhangs of two DNA ends, and then, the non-homologous flaps are removed, and the remaining ssDNA is filled in and ligated. And DNA ends having long-range resection are preferred for homology-directed repair (HDR) pathway, which uses the information from a homologous DNA template to accurately repair double-strand breaks and thus HDR, unlike NHEJ and MMEJ, does not lead to insertions and deletions in the repaired sequence (Xue & Greene 2021; Yao et al. 2017).

The ability to control the structure of Cas9-generated DSBs is a way to alter DNA repair pathway choice and thereby control the outcome of genome editing. Wild-type Cas9 preferentially induces indels even in the presence of the DNA donor template (Miyaoka et al. 2016). There are several reasons for this bias of Cas9-induced DSBs. First, NHEJ, which is responsible for indels, is the predominant repair pathway for double-stranded DNA breaks, and HDR is the least active one (Pannunzio et al. 2018). Second, Cas9 generates predominantly blunt ends (Jiang and Doudna 2017), which serve as a substrate for the NHEJ pathway (Kaminski et al. 2022). Third, Cas9 is also capable of generating staggered ends with 1nt 5′-overhang (Lemos et al. 2018; Müthel et al. 2023; Přibylová et al. 2022), which are also substrates for Polλ operating within the NHEJ pathway (Kaminski et al. 2022) and leading to a single nucleotide insertion into the target sequence. Molecular dynamics simulations confirmed that the ruvC domain has the conformational flexibility to generate 1 nt 5′-overhangs (Zuo and Liu 2016). Moreover, depending on the target sequence, Cas9 can induce DSBs with longer 5′-overhangs (Shi et al. 2019; Shou et al. 2018; Chauhan et al. 2023), indicating significantly greater flexibility of the ruvC domain. Recently, a vCas9 variant carrying a combination of mutations (S55R-R976A-K1003A-T1314R) that promotes the generation of long sticky ends was generated (Chauhan et al. 2023). Long sticky ends suppress NHEJ and direct DSBs repair predominantly through the MMEJ or HDR pathway. The authors suggested that a possible mechanism is to change the position of the non-target DNA strand relative to the ruvC active center. The GFP-to-BFP reporter system showed that although the percentage of cells edited by vCas9 and recovered by HDR doubled, the proportion of cells with inactivated GFP remained the largest (Chauhan et al. 2023). These results suggest that the NHEJ and MMEJ pathways still have a significant influence on the outcome of gene editing. Nevertheless, the vCas9 variant represents a good platform for further engineering Cas9 toward a less mutagenic genome editor.

## Changing the DNA repair outcome: complete inactivation of the HNH domain

A recent study showed that the H840A mutation does not completely inactivate the HNH domain (Lee et al. 2023) as it should be based on previous works (Jinek et al. 2012; Anzalone et al. 2019). As a result, Prime Editor with mutated HNH (H840A) can still induce unwanted DSBs, resulting in gene-disruptive indels with an average frequency of 2.5 ± 0.6%. Introduction of N863A in addition to the H840A mutation significantly reduces the ability of double-mutant NHN to induce DSBs to 0.34 ± 0.06%. To further reduce the activity of the HNH domain, the authors examined combinations of other residues in the active center based on the Cas9 structure in the cleavage state (PDB ID 6O0Y) (Zhu et al. 2019). They identified three combinations of H840A + N854A, H840A + N863A + N854A, and 840A + N863A + D839A + N854A mutations that reduce DSBs-producing activity to 0.02 ± 0.01% (Lee et al. 2023). The results of this work also warn that the dCas9 mutant, which is widely used in artificial transcription factors and other epigenetic editors (Brocken et al. 2018), may still have some nuclease activity that negatively affects the activity of these editors. There is no doubt that successfully engineered Cas9 variants should replace the currently used nCas9 and dCas9 mutants.

## Reducing the immunogenicity of Cas9 by eliminating immunogenic epitopes

**Fig. 6:**
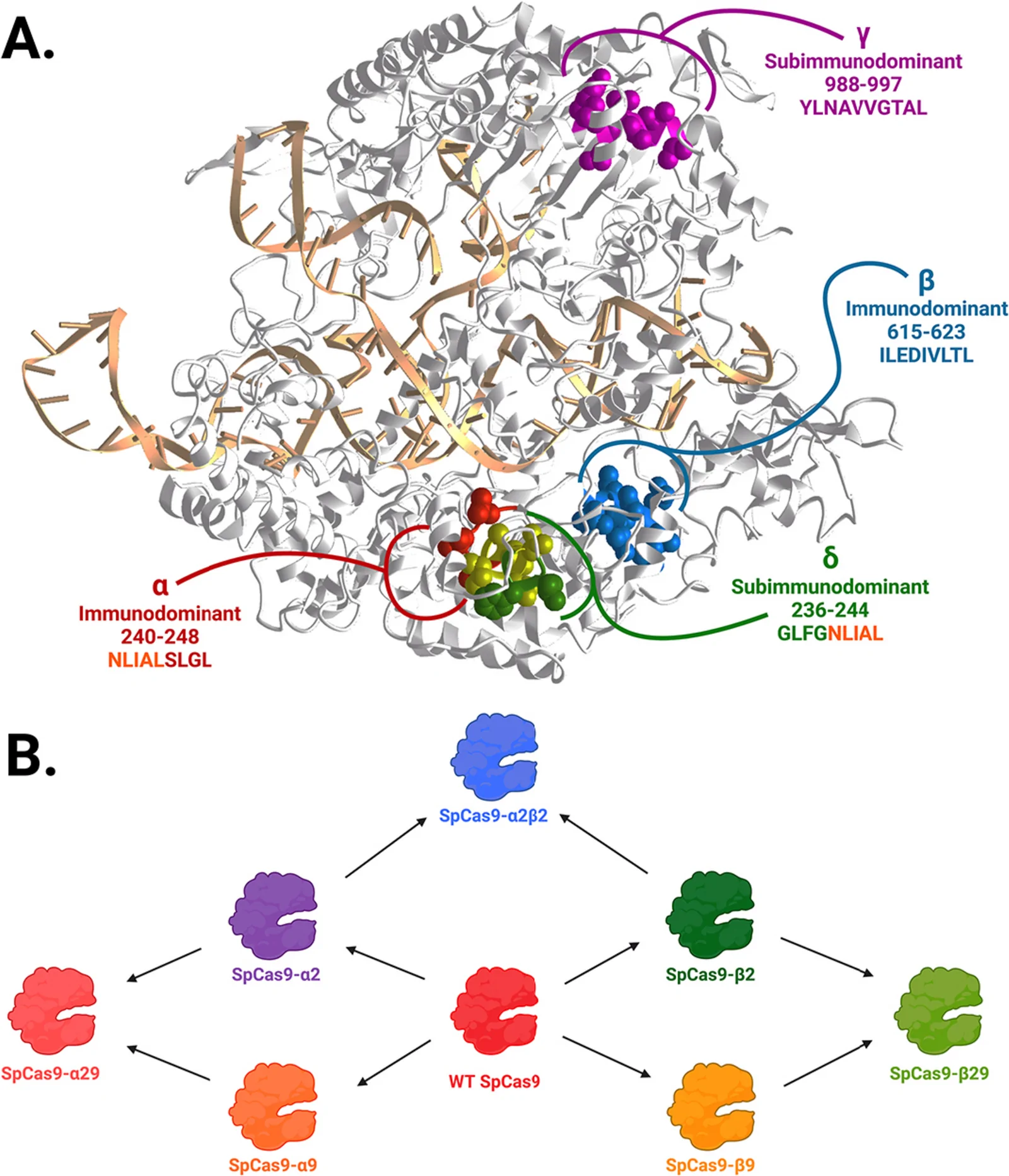
Less immunogenic Cas9 variants. **a**. Positions of immunogenic peptides in the Cas9 structure. The structure is PDB ID 4UN3 (Anders et al. 2014). Epitopes α, β, γ, and δ are shown in red, blue, purple, and green, respectively; the intersection between α and δ is shown in yellow; colors are chosen according to the esthetic preferences of the authors. The initial picture was created using icn3d (Wang et al. 2022). The final version was created with BioRender.com. **b**. Development of Cas9 variants with inactivated epitopes. Created with BioRender.com

Cas9 as a foreign protein is capable of inducing a humoral and cytotoxic T-cell immune response in humans, dogs, and other animals, which leads to a decrease in its therapeutic effectiveness (Ferdosi et al. 2019; Charlesworth et al. 2019; Hakim et al. 2021). According to some authors, immunosuppression can help in vivo (Gillmore et al. 2021), while in other works immunosuppression as well as the use of tissue-specific promoters are ineffective (Hakim et al. 2021). Therefore, one of the important directions of Cas9 engineering is the creation of its less immunogenic forms. Immunodominant (α, 240–248 aa; β, 615–623 aa) and subimmunodominant epitopes (γ, 988–997 aa; δ, 236–244 aa) were localized primarily in the REC lobe (Ferdosi et al. 2019) (Fig. 6). Mutations of MHC-binding anchor residues in the α-epitope (L241G, L248G, L241G + L248G) and in the β-epitope (L616G, L623G, L616G + L623G) reduced protein immunogenicity by more than an order of magnitude. Moreover, at least SpCas9-α2 and SpCas9-β2 have activity comparable to the wild-type protein. Mutations in the REC domains are frequently found in high-fidelity Cas9 variants (Table 1), and the authors checked the off-target rates of SpCas9-β2 variant. To do so, they used it in the form of epigenetic activator and subsequent RNA-seq showed no significant off-targets. Therefore, specificity of immunomodified Cas9 variants as DNA nucleases currently is unknown.

## Conclusions and future perspectives

The first generation of Cas9 editors revealed the costs of high specificity, which began to be overcome in the next-generation editors. Existing variants show that it is possible to create Cas9 editors with increased specificity while maintaining high on-target activity, increased target range, low mutagenicity, and low immunogenicity. These variants can be used as platforms for further improvement and to explore the possibility of combining mutations to create Cas9 editors with an expanded spectrum of therapeutic applications.

## Data Availability

All data supporting the findings of this study are available within the paper.
